# Simulation Modeling of Reduced Glycosylation Effects on Potassium Channels of Mouse Cardiomyocytes

**DOI:** 10.3389/fphys.2022.816651

**Published:** 2022-03-04

**Authors:** Haedong Kim, Hui Yang, Andrew R. Ednie, Eric S. Bennett

**Affiliations:** ^1^Complex Systems Monitoring, Modeling, and Control Laboratory, The Pennsylvania State University, University Park, PA, United States; ^2^Department of Neuroscience, Cell Biology, and Physiology, Wright State University, Dayton, OH, United States

**Keywords:** N-glycosylation, dilated cardiomyopathy, potassium channel, simulation modeling, genetic algorithm

## Abstract

Dilated cardiomyopathy (DCM) is the third most common cause of heart failure and the primary reason for heart transplantation; upward of 70% of DCM cases are considered idiopathic. Our *in-vitro* experiments showed that reduced hybrid/complex N-glycosylation in mouse cardiomyocytes is linked with DCM. Further, we observed direct effects of reduced N-glycosylation on K_v_ gating. However, it is difficult to rigorously determine the effects of glycosylation on K_v_ activity, because there are multiple K_v_ isoforms in cardiomyocytes contributing to the cardiac excitation. Due to complex functions of K_v_ isoforms, only the sum of K^+^ currents (I_Ksum_) can be recorded experimentally and decomposed later using exponential fitting to estimate component currents, such as I_Kto_, I_Kslow_, and I_Kss_. However, such estimation cannot adequately describe glycosylation effects and K_v_ mechanisms. Here, we propose a framework of simulation modeling of K_v_ kinetics in mouse ventricular myocytes and model calibration using the *in-vitro* data under normal and reduced glycosylation conditions through ablation of the Mgat1 gene (i.e., Mgat1KO). Calibrated models facilitate the prediction of K_v_ characteristics at different voltages that are not directly observed in the *in-vitro* experiments. A model calibration procedure is developed based on the genetic algorithm. Experimental results show that, in the Mgat1KO group, both I_Kto_ and I_Kslow_ densities are shown to be significantly reduced and the rate of I_Kslow_ inactivation is much slower. The proposed approach has strong potential to couple simulation models with experimental data for gaining a better understanding of glycosylation effects on K_v_ kinetics.

## 1. Introduction

Heart disease is the leading cause of death globally, accounting for 23% of deaths in the U.S. in 2017 (Heron, 2019). Dilated cardiomyopathy (DCM) is the third most common cause of heart failure and the most frequent reason for heart transplantation (Weintraub et al., 2017). DCM is characterized by enlarged and weakened ventricular chambers, and it is associated with systolic and contractile dysfunction that has a high risk to heart failure, with approximately 70% of DCM cases regarded as idiopathic (Hershberger and Siegfried, 2011; Lakdawala et al., 2013; Weintraub et al., 2017). There has been consistent and increasing evidence of a link between aberrant glycosylation and heart failure (Gehrmann et al., 2003; Footitt et al., 2009; Marques-da Silva et al., 2017). Recently, we showed that reduction of hybrid/complex N-glycosylation in mouse cardiomyocytes, through ablation of the Mgat1^1^ gene that encodes a critical glycosyltransferase (GlcNAcT1^2^) (Mgat1KO model), is sufficient to cause DCM (Ednie et al., 2019a,b). Mgat1KO mice develop DCM, heart failure, and 100% die early, likely from ventricular arrhythmias resulting in sudden cardiac death. Further, Mgat1KO ventricular myocytes demonstrated altered electromechanical functions, including excitation-contraction (EC) coupling, are consistent with observed changes in electrical signaling caused by acute and downstream (disease-related) effects on voltage-gated ion channel (VGIC) gating and activity (Ednie et al., 2019b).

We investigated the impact of reduced hybrid/complex N-glycosylation through the lens of electrophysiology. Electrical signaling in the heart has vital functions related to intra and extracellular communication, rhythmicity of heartbeats, and provides a driving force for contraction (Koenig et al., 2011). This electrical stimulation is called the action potential (AP), which is the net transmembrane potential varying over time.

**Figure 1:**
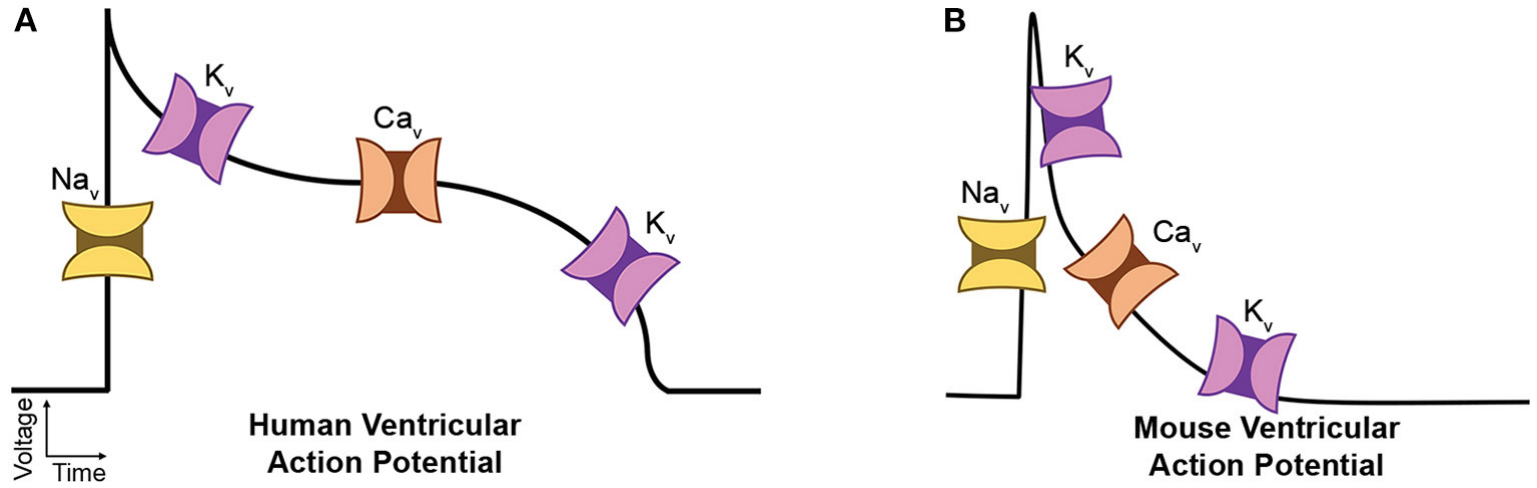
Diagrams of major cardiac ion channels and their roles in the action potential. **(A)** Human ventricular myocytes. **(B)** Mouse ventricular myocytes.

The AP is the result of orchestrated activities of various underlying ionic currents through VGICs (Grant, 2009). Even small changes in VGIC function can contribute to aberrant AP waveform and/or conduction, leading to arrhythmias. As illustrated in Figure 1, Na^+^ channels (Na_v_) are responsible for AP initiation, or the depolarization phase. Ca^2+^ channels (Ca_v_) drive the prolonged depolarization phase particularly in larger species such as human (“plateau”) as in Figure 1A, while in small species, the AP re-polarizes rapidly without the long duration of intracellular Ca^2+^ flux as in Figure 1B because smaller contractile force is required. Several K^+^ channel (K_v_) isoforms are collectively responsible for AP deactivation, i.e., re-polarization. The human ether-a-go-go gene (hERG) K_v_ is mainly responsible for the rapid re-polarization after the plateau phase. However, mouse ether-a-go-go gene (mERG) is almost absent in the mouse ventricular AP, where the re-polarization is generally delineated as kinetic components of a rapidly inactivating transient outward current and delayed rectifier-type currents. Like most transmembrane proteins, VGICs are heavily glycosylated membrane proteins (Ednie and Bennett, 2011). Studies have proven that glycosylation can affect VGIC function predominantly. For example, we reported that a saturating, electrostatic effect of negatively charged sialic acids, which are typically attached to the terminal of glycan branches, significantly altered cardiac electrical signaling (Ednie et al., 2013; Ednie and Bennett, 2015). Our recent studies observed aberrant electrical signaling, e.g., prolonged APs and abnormal early re-activations, in Mgat1KO ventricular apex myocytes (Ednie et al., 2019a,b). These observations strongly support direct and disease-related effects of reduced N-glycosylation on VGIC gating and activity.

Although these *in-vitro* experiments showed changes in activities of ion channels, there are certain limitations that include: (1) *Detailed ion channel kinetics are difficult to determine rigorously using in-vitro experiments alone*. From the electrophysiological experiment, pathological electrical signaling can be observed through measurements of APs at the whole-cell level and currents at the ion-channel level. However, it is difficult to relate, rigorously, the impacts of VGIC gating changes to the altered AP waveform/conduction and *vice versa*. (2) *Segregating different K*^+^ *currents is difficult using whole-cell recording experimental techniques only*. I_Ksum_ is the result of joint activity of multiple isoforms with each isoform producing a different, but slightly overlapping (in activation and inactivation voltages) component of the total K^+^ current (I_Ksum_) that contributes to various portions of AP re-polarization (Du et al., 2018). However, *in-vitro* experiments can only measure the sum of these component currents (I_Ksum_) using whole-cell voltage-clamp protocols. Even pharmacologic separation of K_v_ current types is difficult, as the specificity of drugs for a single current type is not ideal. Hence, component K^+^ currents are usually estimated through multiple exponential fits of the decaying portion (due to channel inactivation) of the total K^+^ current (Brunet et al., 2004). Although the exponential function illustrates the shape of a component-current trace well, it cannot adequately describe kinetic dynamics of the current, nor fully and rigorously distinguish among currents produced by different K_v_ isoforms because of their slightly overlapping voltage-dependence of gating. As K_v_ have a critical function in re-polarizing the cell, it is imperative to model and examine K_v_ isoforms thoroughly.

Therefore, this article presents simulation modeling of dominant K^+^ currents in mouse ventricular myocytes and further calibrate their parameters using the *in-vitro* data under normal and reduced glycosylation conditions through ablation of the Mgat1 gene. Specifically, we propose an optimization procedure to calibrate simulation models of potassium channel isoform activity, I_Kto_. Key statistics of component currents, amplitudes, and inactivation rates (τ's) are measured from *in-vitro* experimental data with a 4.5 s voltage-clamp protocol (see details in *in-vitro* Experimental Data Section in Material and Methods). The major contributions of this article are summarized as follows.

- Simulation models are integrated with *in-vitro* experiments to investigate the effects of reduced N-glycosylation on K_v_ activity of mouse myocytes. Computer simulation helps demonstrate the voltage-gating mechanism and conductance kinetics that cannot be readily available through traditional exponential fitting.
- Calibrated models facilitate the prediction of K_v_ characteristics at different voltages that are not observed in the *in-vitro* experiments. In the Mgat1KO group, both I_Kto_ and I_Kslow_ densities are shown to be significantly reduced and the rate of I_Kslow_ inactivation is much slower.
- Simulation modeling of cardiac myocytes is conducive to gain a better understanding of detailed K_v_ kinetics, as well as how reduced glycosylation through ablation of Mgat1 gene impacts K_v_ kinetics.

## 2. Materials and Methods

### 2.1. Glycosylation and Dilated Cardiomyopathy (DCM)

Protein glycosylation is an essential cellular process that impacts many cell functions (Marques-da Silva et al., 2017). Briefly, protein glycosylation is the sequential co-/post-translational process of attaching sugar residues (glycans) to proteins. Cardiac VGICs are heavily glycosylated proteins with upwards of 30% of their mass consisting of N- and O-linked glycans. A growing number of cardiac diseases, including DCM and hypertrophic cardiomyopathy, can present with concurrent, albeit, modest changes in glycosylation (Gehrmann et al., 2003; Footitt et al., 2009; Marques-da Silva et al., 2017). Mgat1 expression was implicated in cardiac function and shown to be down-regulated in human end-stage idiopathic DCM. Genome-wide searches identified changes in glycosylation-related gene expression in human idiopathic DCM, including glycosyltransferases (Barrans et al., 2002; Hwang et al., 2002; Yung et al., 2004); and proteomic/glycomic studies show changes in serum N-glycosylation in heart disease models and in humans with DCM risk factors (Nishio et al., 1995; Knezevic et al., 2009; Yang et al., 2015; Miura and Endo, 2016; Nagai-Okatani and Minamino, 2016). Models of DCM/heart failure were associated with subtle changes in glycosylation of proteins involved in electromechanical processes, and 20% of patients with congenital disorders of glycosylation (CDG) present with cardiac deficits, including idiopathic DCM (Gehrmann et al., 2003; Marques-da Silva et al., 2017). The data suggest a correlation between modest changes in extracellularly facing glycosylation and DCM/heart disease.

### 2.2. Potassium Currents Analysis and *in-vitro* Experimental Data

**Figure 2:**
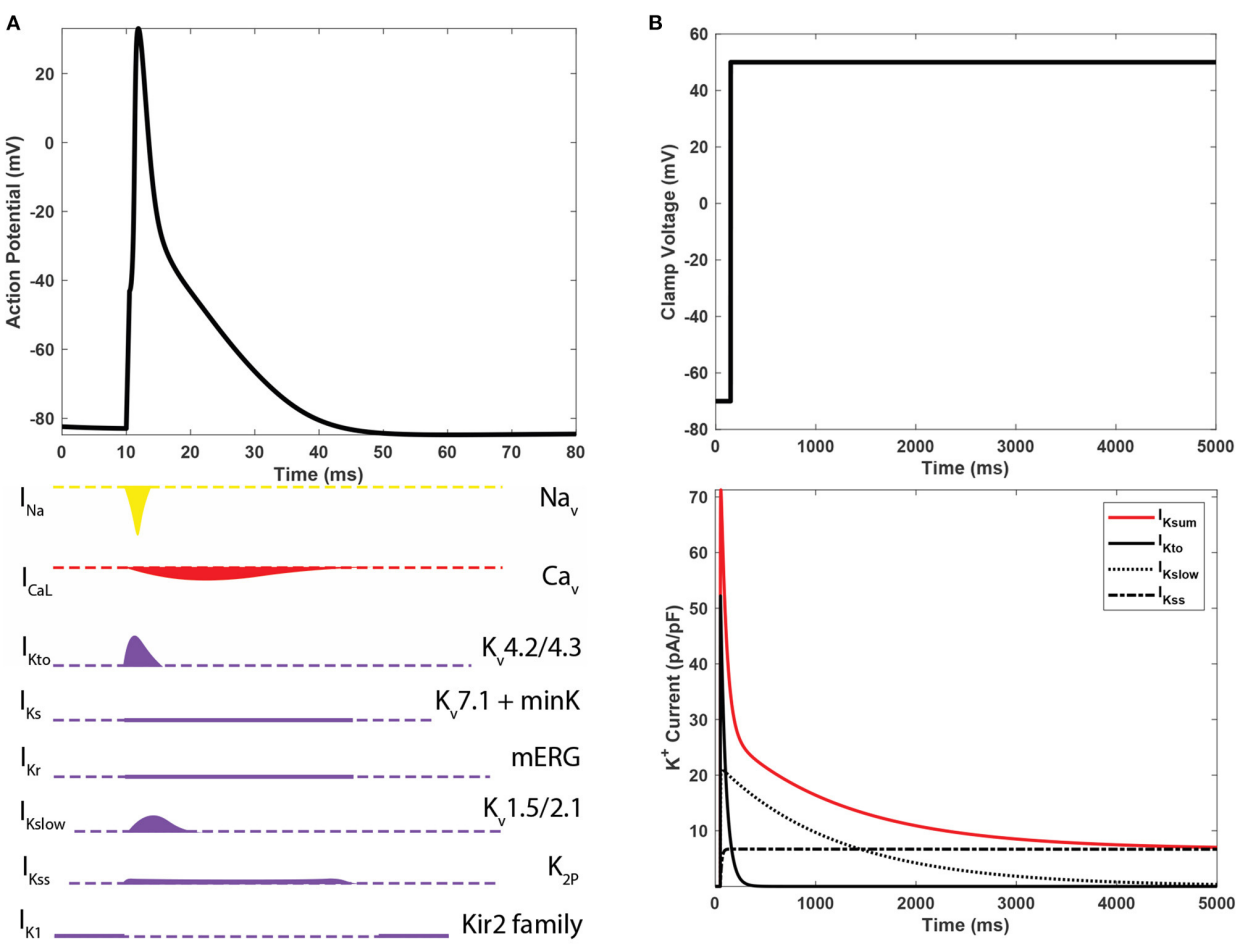
**(A)** The action potential of mouse ventricular myocytes and underlying ioninc currents. **(B)** Predominant K^+^ currents in mouse cardiomyocytes and their shapes of whole-cell voltage clamp recordings.

The orchestrated activity of different K_v_ isoforms produces unique but slightly overlapping currents, as illustrated in Figure 2A. However, it is difficult to rigorously delineate the multiple component K^+^ currents through voltage-clamp experiments alone because only the sum of K^+^ currents is measured and recorded. I_Ksum_ is usually decomposed mathematically by fitting the peak and decaying portion of each component current trace with an exponential function (Brunet et al., 2004). A standard exponential function, I = A*e*^−*t*/τ^+C has three parameters: amplitude A, time constant τ, and constant offset value C. *t* is time in millisecond. Amplitude refers to the value of the peak and τ to the time constant when current reduces by *e*^−1^ ( 63%) of the peak.

As illustrated in Figure 2B, I_Ksum_ is often decomposed into three dominant currents: a rapidly inactivating transient outward current (I_Kto_), a delayed rectifier-type current (I_Kslow_), and a non-inactivating steady-state current (I_Kss_) (Nerbonne and Kass, 2005). I_Kto_ has a high peak at the very beginning of activation and rapid inactivation. I_Kslow_ has a low peak and longer inactivation phase. I_Kss_ remains constant during the course of depolarization. A bi-exponential function with a constant component, as in Equation (1), is a mathematical form for decomposing I_Ksum_ into the three major K^+^ currents. In Equation (1), A_*Kto*_ and A_*Kslow*_ are the amplitudes; τ_*Kto*_ and τ_*Kslow*_ are time constants of I_Kto_ and I_Kslow_, respectively. A_*Kss*_ is the constant current I_Kss_. Although bi-exponential fitting captures essential characteristics of three major component K^+^ currents and describes the I_Ksum_ waveform, it cannot provide detailed kinetic dynamics of K_v_ isoforms (Plumlee et al., 2016).


(1) $$\begin{array}{r} I_{\text{Ksum}}={\text{A}}_{Kto}e^{-t/\tau_{Kto}}+{\text{A}}_{Kslow}e^{-t/\tau_{Kslow}}+{\text{A}}_{Kss} \end{array}$$

Recently, we reported electrophysiological experiment data to investigate the impact of reduced hybrid/complex N-glycosylation on left ventricular cardiomyocyte activity, through deletion of the Mgat1 gene, which encodes a critical glycosyltransferase (GlcNAcT1) (Ednie et al., 2019a,b). The detailed process of creation of Mgat1KO (Mgat1 Knock Out) strain, features of the cardiomyocyte-specific Mgat1KO strain, breeding, genotyping, and selection of wild type (WT) animals were previously described in Ednie et al. (2019a). In our previous study of K_v_ activities in the Mgat1KO (Ednie et al., 2019b), cells of 12 to 20-week-old mice were used and numbers of observation for WT and Mgat1KO are 35 and 38, respectively.

**Figure 3:**
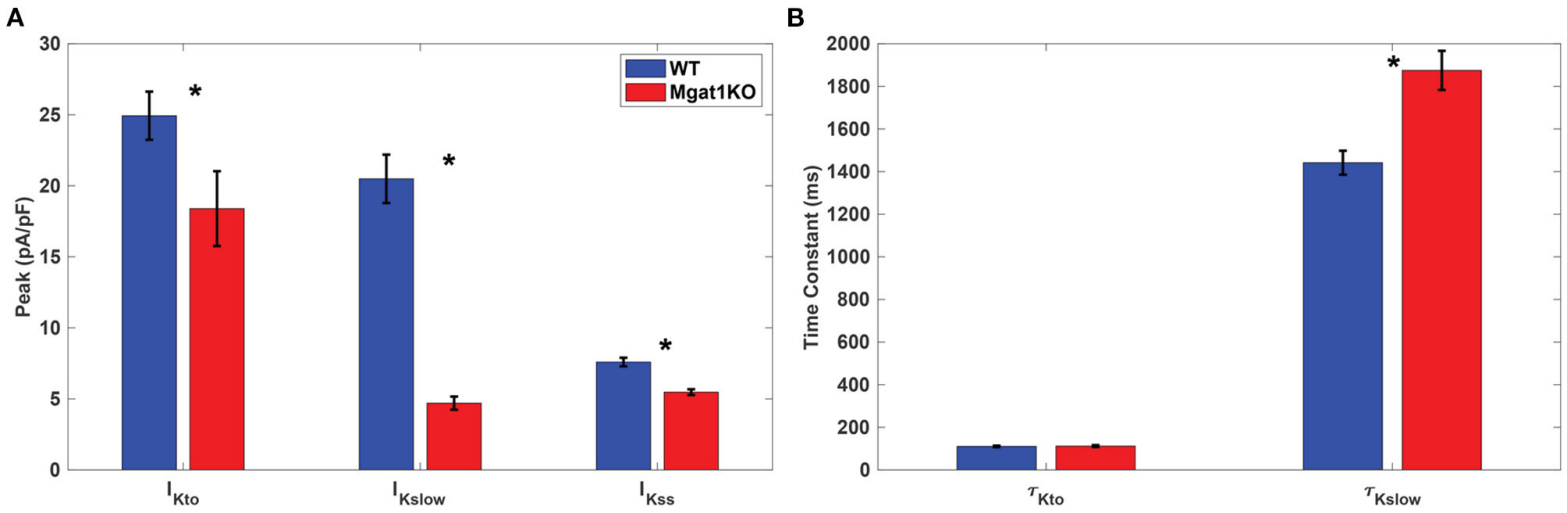
*In-vitro* experimental results, adopted from Ednie et al. (2019b). **(A)** Averages and error bars of the peaks of I_Kto_ and I_Kslow_, and steady-state amplitude of I_Kss_. **(B)** Inactivation time constant of I_Kto_ and I_Kslow_. Significant differences between WT and Mgat1KO at *p* ≤ 0.05 are indicated by an *. (*n* = 35 for WT and *n* = 38 for Mgat1KO).

I_Ksum_ was measured using whole-cell voltage clamp protocols. Cells were held at -70 mV and then depolarized by 10 mV voltage steps from -50 to 50 mV for 4.5 s. Figure 3 shows our *in-vitro* experimental data about key statistics on I_Kto_ and I_Kslow_. As shown in Figure 3A, peaks of both I_Kto_ and I_Kslow_ are reduced in the Mgat1KO group; and steady-state current I_Kss_, remaining constant during the depolarization, is also reduced in the Mgat1KO group. A_*Kslow*_ is most reduced compared to WT, by 77%. Figure 3B compares the time constants τ. τ_*Kslow*_ of the Mgat1KO group is 30% larger than the WT group, which means I_Kslow_ is prolonged with reduced N-glycosylation. However, τ_*Kto*_ does not show significant difference.

### 2.3. *In-silico* Simulation Modeling

The AP is modeled as an orchestrated activity of various ionic currents as given in Equation (2). In this equation, *C*_*m*_ is the membrane capacitance, and there are several ionic currents: L-type calcium current (I_CaL_), calcium pump current (I_p(Ca)_), Na^+^/Ca^2+^ exchange current (I_NaCa_), calcium background current (I_Cab_), fast Na^+^ current (I_Na_), background Na^+^ current (I_Nab_), Na^+^/K^+^ pump current (I_NaK_), fast transient outward K^+^ current (I_Kto, f_), slower transient outward K^+^ current (I_Kto, s_), which is essentially missing in apex myocytes, time independent K^+^ current (I_K1_), slow delayed rectifier K^+^ current (I_Ks_), ultrarapidly activating delayed rectifier K^+^ current (I_Kur_), non-inactivating steady-state K^+^ current (I_Kss_), rapidly delayed rectifier K^+^ current (I_Kr_), Ca^2+^-activated Cl^-^ current (I_Cl, Ca_), and stimulus current (I_stim_). Although there are seven currents pertinent to K_v_ dynamics in this model, as mentioned earlier, three current types, I_Kto_, I_Kslow_, and I_Kss_, are dominant K^+^ currents in a ventricular apex myocyte (also see Figure 2A). Thus, in this study, we focus on development of computer models of I_Kto_ and I_Kslow_. I_Kss_ is assumed as a constant as in Equation (1). This model can be flexibly modified to include I_Kto, s_ for the septum. The details of ionic currents models and their kinetics can be found in Bondarenko et al. (2004).


(2) $$\begin{array}{c} -C_{m}\frac{dV}{dt}={\text{I}}_{\text{CaL}}+{\text{I}}_{\text{p}\left(Ca\right)}+{\text{I}}_{\text{NaCa}}+{\text{I}}_{\text{Cab}}\\ \;+{\text{I}}_{\text{Na}}+{\text{I}}_{\text{Nab}}+{\text{I}}_{\text{NaK}}+{\text{I}}_{\text{Kto},f}\\ \;+{\text{I}}_{\text{Kto},s}+{\text{I}}_{\text{K}1}+{\text{I}}_{\text{Ks}}+{\text{I}}_{\text{Kur}}+{\text{I}}_{\text{Kss}}+{\text{I}}_{\text{Kr}}\\ \;+{\text{I}}_{\text{Cl},Ca}+{\text{I}}_{\text{stim}} \end{array}$$

There are two major modeling schemes for VGICs: Markov models and Hodgkin-Huxley models. While Markov modeling of ionic currents is popular such as O'Hara models (O'Hara et al., 2011; O'Hara and Rudy, 2012), Hodgkin-Huxley modeling is commonly used to formulate K^+^ currents for various species, for example, not only for mouse ventricular myocytes (Bondarenko et al., 2004) but also for rabbit (Mahajan et al., 2008) and human ventricular myocytes (ten Tusscher et al., 2004). I_Kto_ and I_Kslow_ are designed using Hodgkin-Huxley modeling as given in Equations (3) and (4), respectively, based on Bondarenko et al. (2004). Hodgkin-Huxley models have two state variables for describing activation and inactivation. In Equation (3), *a*_*to*_ and *i*_*to*_ are two state variables of activation and inactivation, respectively. *G*_*to*_ is the maximum conductance of K_v_4.2, and (*V*−*E*_*K*_) is the difference of voltage *V* and the K^+^ Nernst potential *E*_*K*_. Similarly, the I_Kslow_ model is given as Equation (4), which also include two state variables for activation and inactivation gates. Relevant parametric equations of transition rates and time constants for the gating mechanisms are given below for each current equation.


$$\text{​​​​}{\text{I}}_{\text{Kto}}=G_{to}a_{to}^{3}i_{to}(V-E_{K})\;(3)\;{\text{I}}_{\text{Kslow}}=G_{r}a_{r}i_{r}(V-E_{K})\;(4)$$

$$\begin{array}{l} \frac{da_{to}}{dt}=\alpha_{a}(1-a_{to})-\beta_{a}a_{to}\;\frac{a_{r}}{dt}=\frac{a_{ss}-a_{r}}{\tau_{a}}\\ \frac{di_{to}}{dt}=\alpha_{i}(1-i_{to})-\beta_{i}i_{to}\;\frac{i_{r}}{dt}=\frac{i_{ss}-i_{r}}{\tau_{i}}\\ \alpha_{a}=0.18064e^{0.03577(V+x_{1})}\;a_{ss}=\frac{1}{1+e^{-(V+x_{1})/x_{2}}}\\ \beta_{a}=0.395e^{-0.06237(V+x_{2})}\;i_{ss}=\frac{1}{1+e^{(V+x_{3})/x_{4}}}\;\\ \alpha_{i}=\frac{0.000152e^{-(V+x_{3})/x_{4}}}{0.067083e^{-(V+x_{5})/x_{4}}+1}\;\tau_{a}=0.493e^{-0.0629V}+x_{5}\\ \beta_{i}=\frac{0.00095e^{(V+x_{6})/x_{7}}}{0.051335e^{(V+x_{6})/x_{7}}+1}\;\tau_{i}=x_{6}-\frac{170}{1+e^{(V+x_{7})/x_{8}}} \end{array}$$

### 2.4. Model Calibration

In the literature, optimization procedures have been proposed to calibrate simulation models with *in-vitro* experimental data. Du et al. (2015) integrates statistical metamodeling with *in-vitro* data to investigate the effects of reduced glycosylation on Na^+^ channels in mouse cardiomyocytes. Also, non-linear optimization methods show promising results for calibrating *in-silico* models of K_v_ channels, e.g., hERG channels (Du et al., 2013) and K_v_ isoforms in mouse cardiomyocytes (Du et al., 2018). Note that characteristic curves such as steady-state activation (SSA), steady-state inactivation (SSI), and time constant of inactivation at different voltage were used in these studies to evaluate goodness-of-fit as an objective function of optimization to minimize discrepancies between simulation results and *in-vitro* experimental data. However, these characteristic curves are not always available. In our Mgat1KO data, currents were too small at early-activating voltages to reliably calculate the characteristic curves.

This article presents a new method that calibrates the simulation models with sparse data when rich information of the characteristic curves are not available. To be specific, the proposed method directly fits models to amplitudes and τ's, which are the most critical characteristics to describe component K^+^ current traces. Equation (5) shows the objective function of the suggested optimization procedure, where $\hat{A_{i}}$ and $\hat{\tau_{i}}$ are estimations of amplitude and time constant from the simulation model. Potassium channels from mouse ventricular myocytes are modeled and parameterized based on partial differential equations. Then, full factorial designs are used to discover sensitive variables from the potential set of parameters. Further, a genetic algorithm-based heuristic optimization method is developed to calibrate the models to the data.


(5) $$\begin{array}{r} \min\left(\left|{\text{A}}_{i}-\hat{{\text{A}}_{i}}|+|\tau_{i}-\hat{\tau_{i}}\right|\right),\;i\in \left\{\text{Kto},\text{ Kslow}\right\} \end{array}$$

In *in-silico* experiments, it is critical to identify a sparse subset of sensitive variables (or model parameters to be calibrated) to reduce computational burden and improve modeling accuracy. The curse of dimensionality causes the dramatic surge of required computing resources when the number of variables increases and counter-intuitive geometric properties, making the learning procedure difficult. While a sparse subset of variables restricts the model flexibility to fit data, the opposite also has a detrimental effect on modeling accuracy. We used two-level factorial designs to perform sensitivity analysis and screen the variables that impact the model outputs of our interest. Variables were adjusted at two different levels to assess their effects on model outputs and K_v_ characteristics. For I_Kto_, *x*_1_, *x*_2_, *x*_3_, *x*_6_, *x*_7_, and *G*_*to*_ are selected. For I_Kslow_, *x*_1_, *x*_2_, *x*_3_, *x*_4_, *x*_6_, and *G*_*r*_ are selected.

A metaheuristics optimization method is developed based on the genetic algorithm (GA) to calibrate the computer models. GA mimics the evolution of genes inspired by natural selection, and its procedure is described in Algorithm 1 (Reali et al., 2017). In the standard GA, a new population is constructed from superior solutions of the current population and new solutions are reproduced in Breeding step by so-called crossover. The crossover is a process of mixing up elements of superior solutions in the current population so that this process is also called reproduction. This procedure is expected to discover better candidates for combinatorial optimization problems by searching combinations of elite solutions. Although crossover is an intuitive searching method for combinatorial or discrete optimization problems, exploring combinations is not appropriate for continuous variables. Hence, in this article, we presented a new method named self-breeding genetic algorithm that reproduces new generations without crossover but directly breeds them from each superior solution as described in Algorithm 2.

**Algorithm 1:** Standard Genetic Algorithm.

**Input:** Stopping criterion, Population size *N*, Number of solutions to be selected *k*
**Output:** Best solution x^*^∈ℝ^*n*^ * Initialization*:
1: Generate a random initial population of size *N* *LOOP Process*
2: **while** Satisfying stopping criterion **do**
3: Fitness - Evaluate fitness of each solution in current population
4: Selection - Select *k* solutions with highest fitness and update best solution x^*^
5: Breeding - Generate additional *N*−*k* new solutions by doing crossover elements of top *k* solutions randomly
6: Mutation - Add random noise
7: Update current population with solutions generated through Step 4 and Step 6
8: **end while**
9: **return** x^*^

**Algorithm 2:** Self-breeding Genetic Algorithm.

**Input:** Tolerance (ϵ_*A*_, ϵ_τ_), Target amplitude and time constant (*A*, τ), Population size *N*, Selection size *k*, Breeding size *s*, Lower bound **l**∈ℝ^*p*^, Upper bound **u**∈ℝ^*p*^
**Output:** Model discrepancy (δ_*A*_, δ_τ_), Solution x^*^∈ℝ^*p*^ * Initialization*:
Construct initial population by generating *N* solutions from *Unif*(**l**, **u**) *LOOP Process*
** while** δ_*A*_ ≥ ϵ_*A*_ and δ_τ_ ≥ ϵ_τ_ **do**
Fitness - Evaluate fitness of each solution in current population
Update δ_*A*_ and δ_τ_
Selection - Select *k* solutions **x_*i*_**, *i* = 1, 2, …, *k*, with highest fitness and update best solution x^*^
Calculate variance $\sigma_{i}^{2}$ of top *k* solutions and calculate pooled variance **σ**_*p*_ using recent *w* recent variances, $\sigma_{i}^{2}$, $\sigma_{i-1}^{2}$, …, $\sigma_{i-w+1}^{2}$
for *i* = 1 to *k* **do**
Generate *s* solutions of $x_{i}+N(0,\sigma^{2})$
**end for**
Update current population with top *k* solutions generated in Step 4 and *ks* solutions generated through Step 6 and Step 8
**end while**
**return** (δ_*A*_, δ_τ_) and x^*^

## 3. Results

The self-breeding GA was applied to a pair of mean values of A_*i*_ and τ_*i*_ in Figure 3, *i*∈{*to, Kslow*}, for each group, WT and Mgat1KO. This calibration process was repeated 30 times. The algorithm stopped when discrepancies of amplitude and time constant were less than tolerances. Tolerances for amplitude and time constant were (0.1, 1.0) for I_Kto_ and (0.1, 5.0) for I_Kslow_, which are smaller than the standard error of mean values. Table 1 shows means and standard errors of calibrated parameters found by the suggested optimization process. The simulation models and optimization process were implemented in MATLAB 2020a, and the codes are available at https://github.com/haedong31/20_Kv_simulation.

**Table 1:** Calibrated model parameters.

I_**Kto**_			I_**Kslow**_		
**Parameter**	**WT**	**Mgat1KO**	**Parameter**	**WT**	**Mgat1KO**
*x*_1_	27.4 ± 4.3	33.9 ± 4.0	*x*_1_	33.2 ± 10.3	18.0 ± 14.0
*x*_2_	42.6 ± 4.8	51.5 ± 7.7	*x*_2_	7.6 ± 0.9	10.7 ± 1.0
*x*_3_	42.5 ± 5.4	48.4 ± 5.2	*x*_3_	54.3 ± 9.0	56.4 ± 7.3
*x*_6_	55.7 ± 2.4	54.7 ± 2.9	*x*_4_	12.4 ± 2.0	10.6 ± 1.3
*x*_7_	36.0 ± 0.8	36.0 ± 1.0	*x*_6_	1432.4 ± 3.1	1853.9 ± 11.5
*G*_*to*_	0.193 ± 0.002	0.142 ± 0.001	*G*_*r*_	0.153 ± 0.001	0.075 ± 0.015

**Figure 4:**
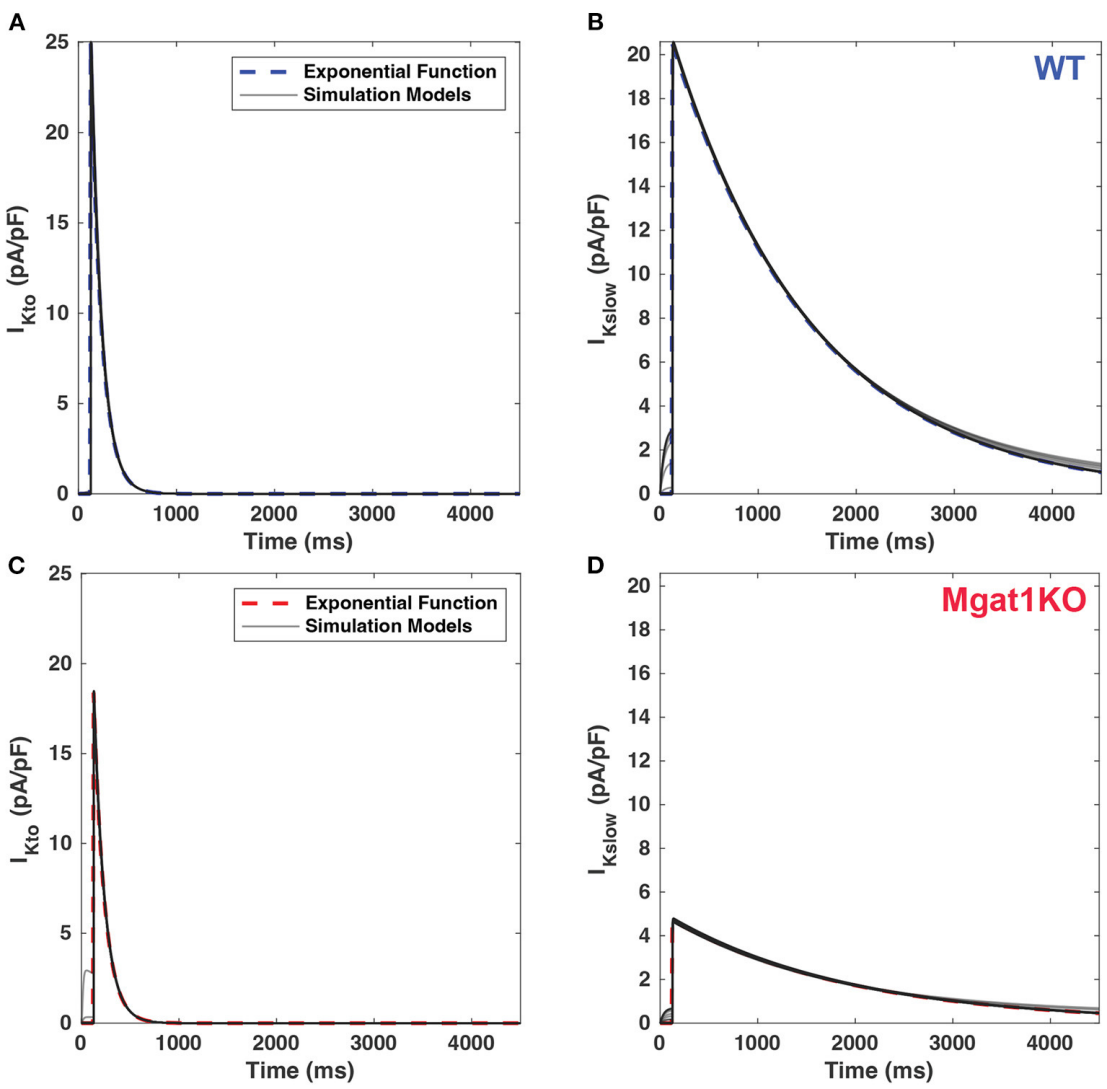
Comparison of current traces between calibrated simulations models (gray line) and benchmark exponential fitting. **(A)** I_Kto_ for WT. **(B)** I_Kslow_ for WT. **(C)** I_Kto_ for Mgat1KO. **(D)** I_Kslow_ for Mgat1KO. Each plot includes 30 replications of simulations results.

Figure 4 shows the current traces generated by the calibrated simulation models and exponential fitting with the mean amplitudes and time constants from *in-vitro* experiment data. Simulation models are shown to be compatible with results of exponential fitting in the electrophysiological data. The solid gray lines represent simulated current traces with 30 repetitions. Except for I_Kslow_ of the Mgat1KO, it is difficult to distinguish the two traces through a visual inspection. For this one, there was a slight dissimilarity during the early decay phase. Also, before applying the clamp voltage, it showed instability that currents had small positive values under the holding potential. Due to the small peak but large time constant, the trace of the Mgat1KO is flattened compared to a standard exponential function, which sharply decreases after the peak. This change is expected to show modeling differences between WT and Mgat1KO.

**Figure 5:**
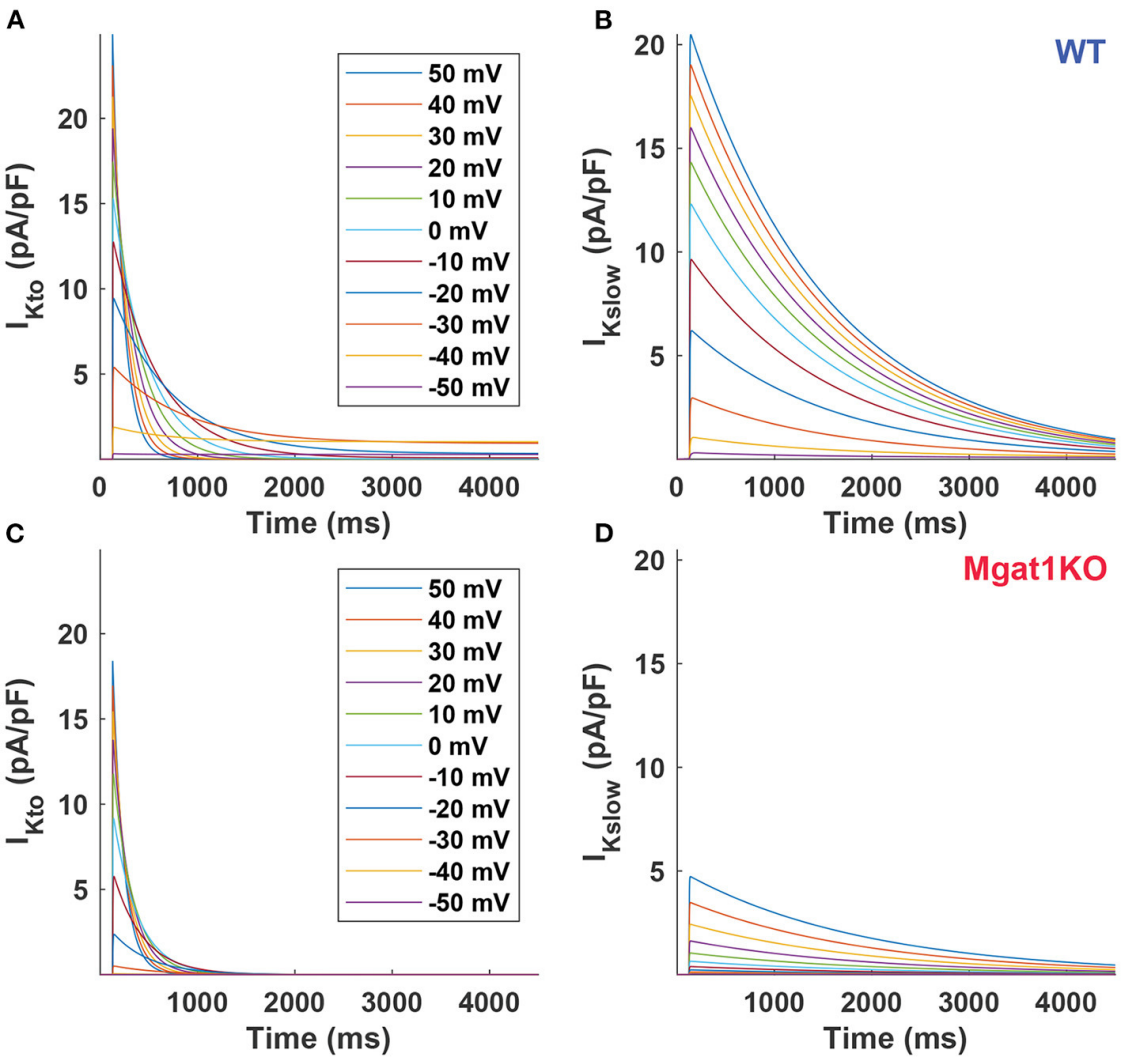
Predicted current traces. **(A)** I_Kto_ for WT. **(B)** I_Kslow_ for WT. **(C)** I_Kto_ for Mgat1KO. **(D)** I_Kslow_ for Mgat1KO. Clamp voltages of 4.5 s were applied to from -50 to +50 mV by 10-mV increments from the holding potential -70 mV.

To further investigate differences between the WT and Mgat1KO groups, current traces were simulated at voltages between -60 and 50 mV in 10-mV increments as shown in Figure 5. Simulated current traces show different patterns as voltage changes between two groups. In Figures 5C,D, the amplitude declines rapidly as voltage decreases for Mgat1KO, while reducing evenly in WT as in Figures 5A,B. Because of the rapid reduction, I_Kslow_ traces for Mgat1KO below 0 mV depolarizations are negligible. In addition, from the perspective of decay phase, the current decreases slowly for I_Kslow_ in the Mgat1KO group. It is predicted that there are differences in conductance values between WT and Mgat1KO.

**Figure 6:**
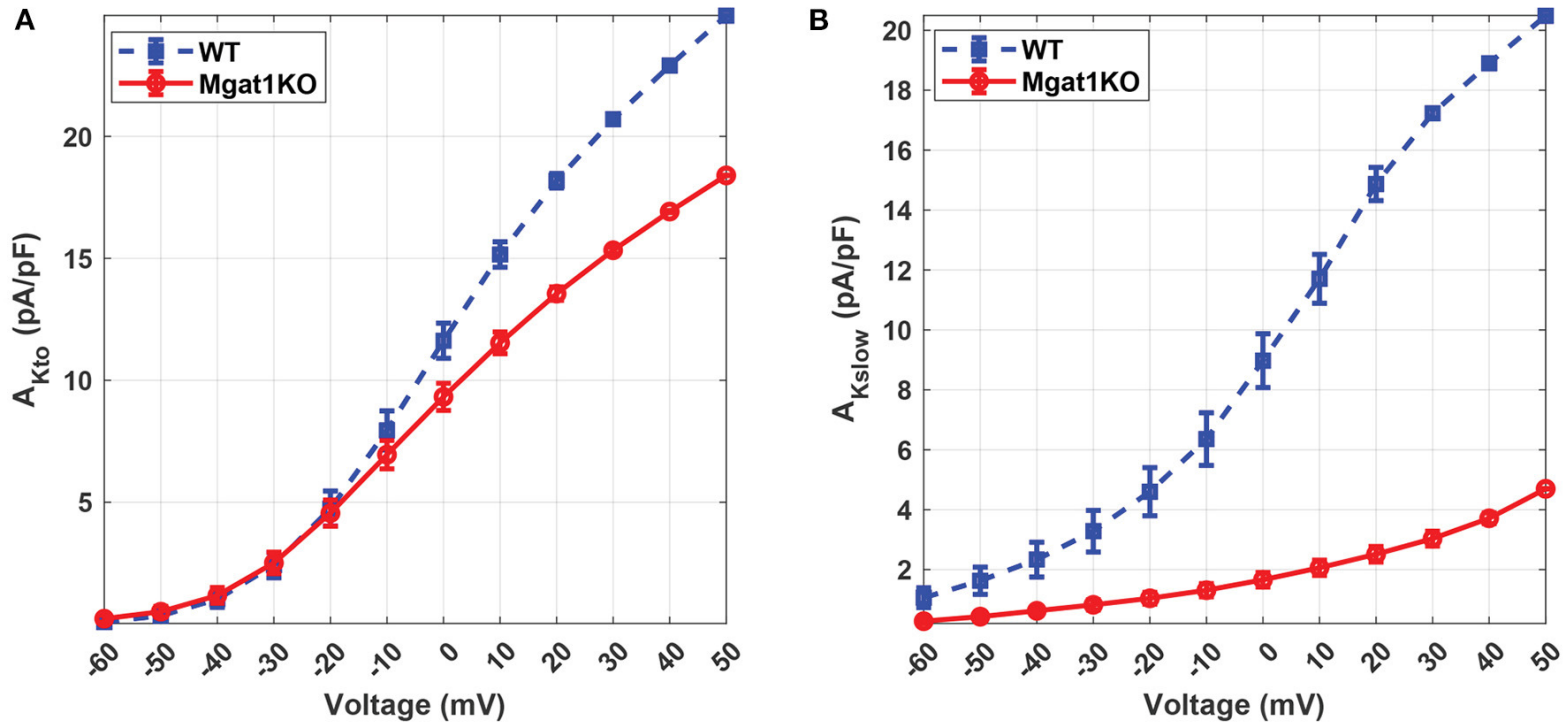
Predicted current density to voltage relationship under the WT and Mgat1KO conditions. **(A)** I_Kto_. **(B)** I_Kslow_. Voltage steps range from -60 to 50 mV by 10-mV increments.

Figure 6 further demonstrates the differences in predicted current density to voltage relationship between WT and Mgat1KO. For both I_Kto_ and I_Kslow_, channels in Mgat1KO are less active at a given depolarized activation voltage compared to WT. The difference in the current peaks between the two groups is more prominent at depolarizations greater than -10 mV. This gap is more significant in I_Kslow_ than I_Kto_. At all voltages, the Mgat1KO I_Kslow_ is smaller than the WT I_Kslow_. This is consistent with *in-vitro* experimental results in which the average amplitude of I_Kto_ and I_Kslow_ were significantly reduced with N-glycosylation perturbation (in the Mgat1KO), and the reduction in Mgat1KO I_Kslow_ was bigger than for Mgat1KO I_Kto_.

**Figure 7:**
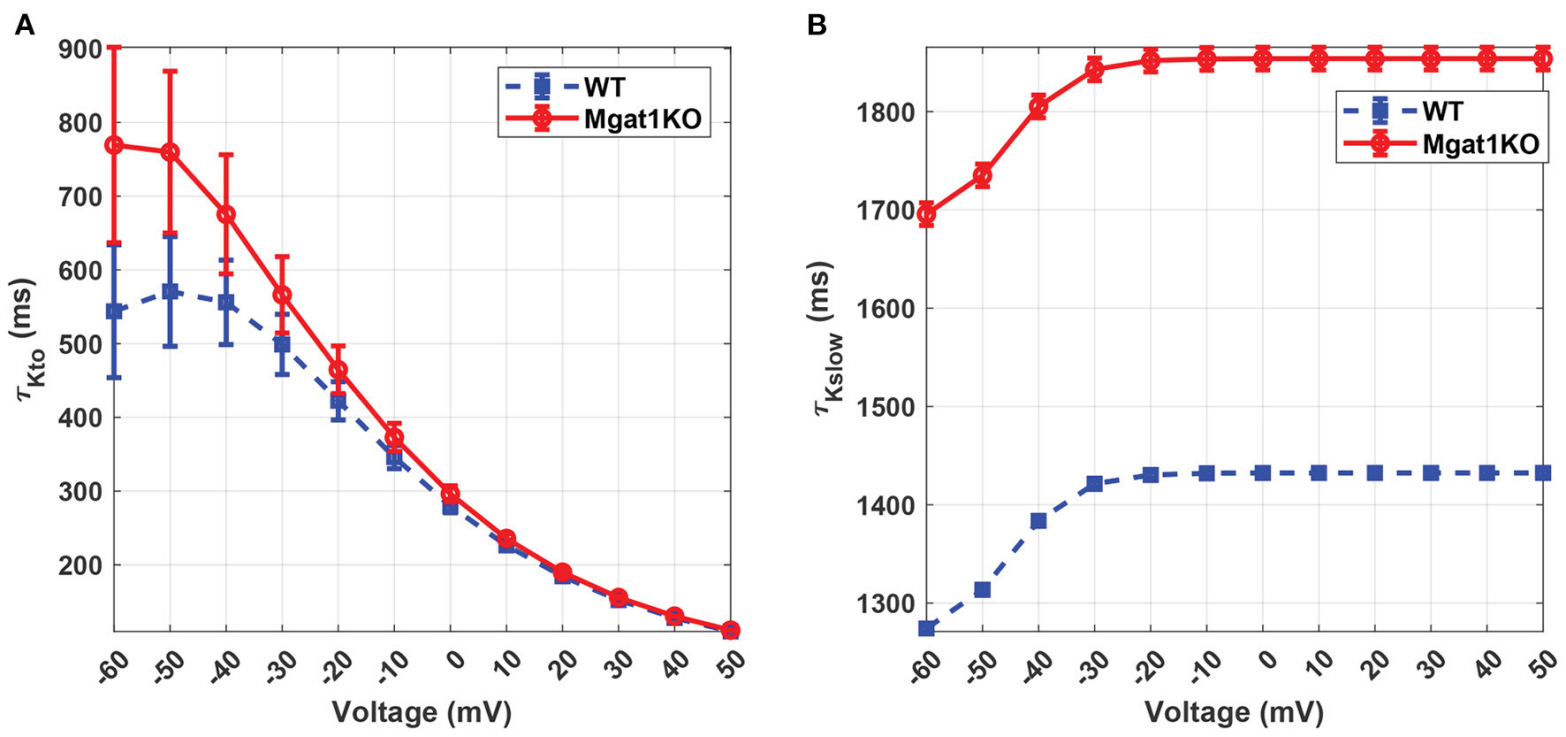
Predicted inactivation time constants to voltage relationship under the WT and Mgat1KO conditions. **(A)** I_Kto_. **(B)** I_Kslow_. Voltage steps range from -60 to 50 mV by 10-mV increments.

**Figure 8:**
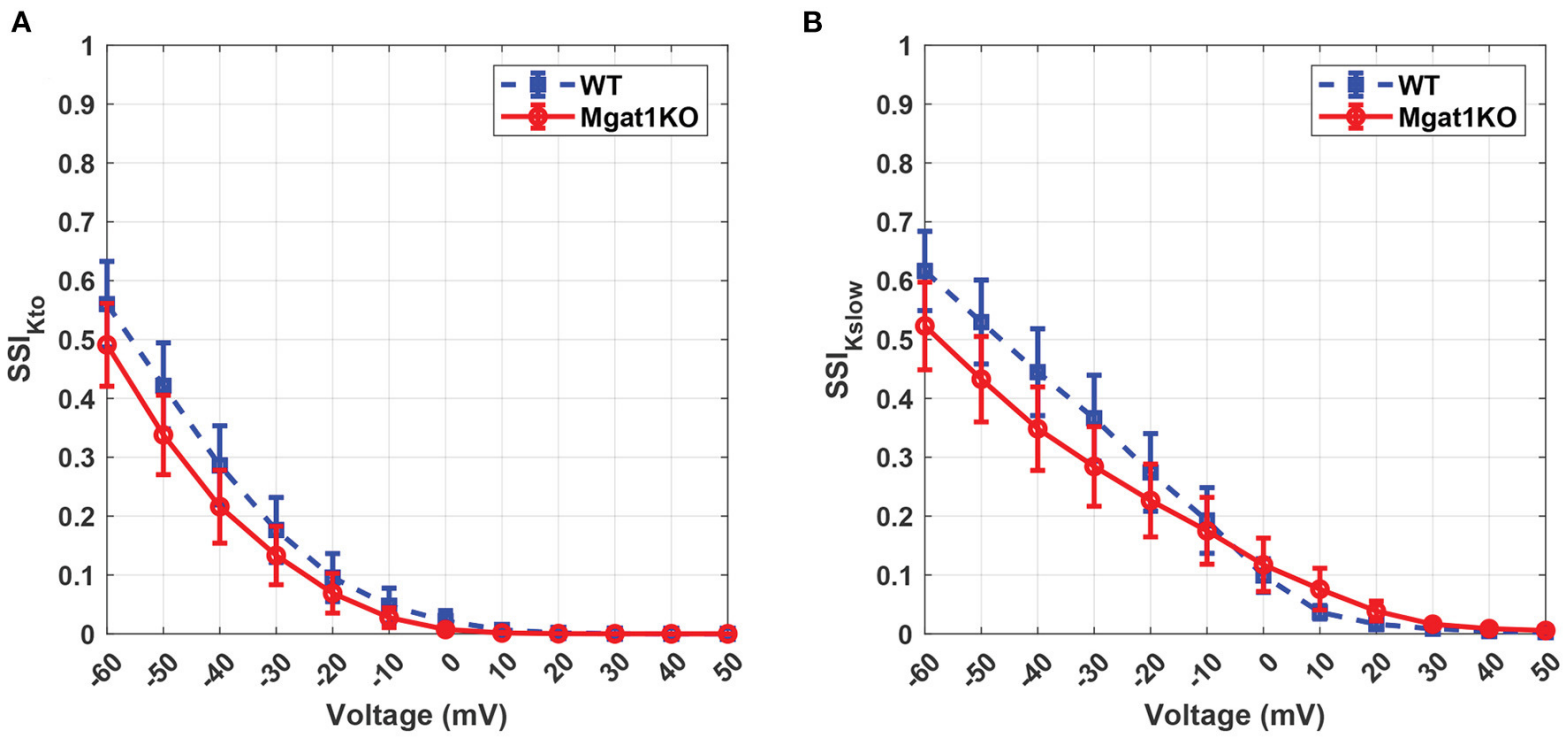
Predicted steady-state inactivation to voltage relationship under the WT and Mgat1KO conditions. **(A)** I_Kto_. **(B)** I_Kslow_. Voltage steps range from -60 to 50 mV by 10-mV increments.

K_v_ has unique inactivation kinetics that makes it difficult to distinguish component K^+^ currents. To investigate its inactivation gating kinetics, inactivation time constants at various voltages were simulated as shown in Figure 7. τ_*Kto*_ does not show a significant difference between Mgat1KO and WT when the depolarization is greater than -20 mV. There are gaps in the case where voltages are less than -20 mV, but the uncertainty represented by the error bars are too wide to make reliable predictions. τ_*Kslow*_ shows consistent differences, with the Mgat1KO inactivating significantly more slowly than WT at all voltages. Figure 8 provides further information of the steady-state inactivation (SSI) rate. The SSI relationships are not significantly different between Mgat1KO and WT for either current type. These data, the longer transition times from open to inactivated state and the lack of a shift in the voltage dependence of K_v_ distribution between open and inactivated states for less glycosylated K_v_ (Mgat1KO), are consistent with our previous studies(Schwetz et al., 2010; Ednie and Bennett, 2015; Du et al., 2018).

**Figure 9:**
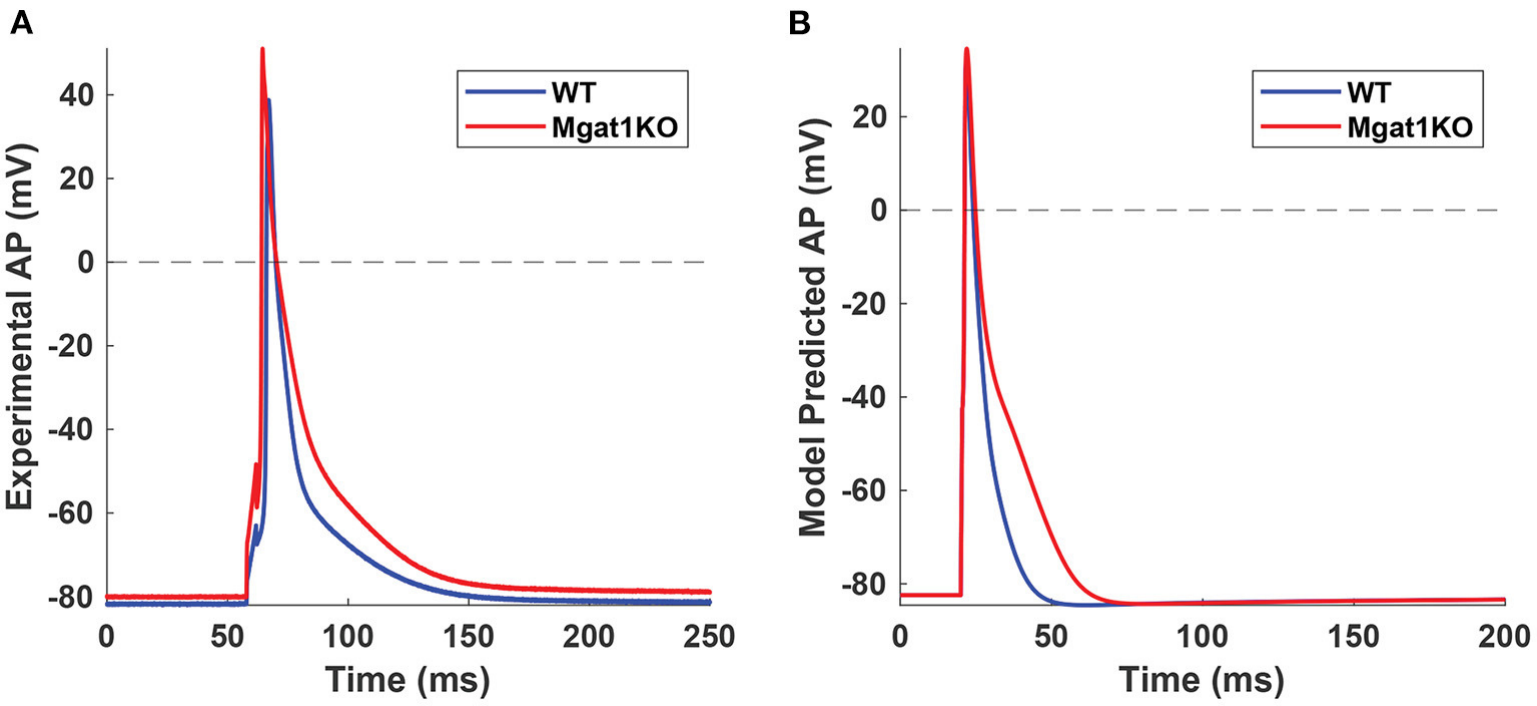
AP traces from WT and Mgat1KO. **(A)** Experimental data (Ednie et al., 2019b). **(B)** Model prediction. APs from the Mgat1KO group are significantly prolonged compared to WT in the repolarization phase.

K_v_ plays a vital role in the repolarization phase. *In-silico* modeling results show a reduction in the magnitudes of both I_Kto_ and I_Kslow_ currents. In particular, I_Kslow_ shows a significant decrease in the current magnitude and a slower inactivation rate. These results are consistent with our experimental data as summarized in Figure 3. Figure 9A shows *in-vitro* experimental data of AP traces from WT and Mgat1KO (Ednie et al., 2019b). Mgat1KO shows prolonged APs in the repolarization phase compared to WT. The magnitude reduction and slow inactivation of I_Kslow_ in Mgat1KO contribute to such AP prolongations. Figure 9B shows the prediction results from our *in-silico* model that are calibrated for I_Kto_ and I_Kslow_ under WT and Mgat1KO conditions. Figure 9B shows how Mgat1KO alters the K^+^ currents and causes the AP to be prolonged. The *in-silico* modeling results corroborate our *in-vitro* experimental data.

## 4. Discussion

This article has developed a self-breeding GA method to calibrate simulation models of K^+^ channel of mouse ventricular apex myocytes based on key statistics and raw data obtained from *in-vitro* voltage-clamp experiments (Ednie et al., 2019b). *In-silico* simulation models allow for the investigation of underlying dynamics of observed current and to make inferences about different experimental conditions that were not yet or cannot be conducted.

Notably, ventricular myocytes consist of several K_v_ isoforms that lead to multiple component K^+^ currents. These component currents collectively produce the total I_Ksum_ that can be recorded during *in-vitro* experiments. Typically, the sum of potassium currents is approximated by fitting multiple exponential functions, each of which is used to estimate the component current. However, traditional exponential functions cannot adequately describe detailed kinetics.

Simulation models were calibrated by comparing their simulated current traces with those derived from the exponential decomposition of I_Ksum_. The simulation models enable the prediction of K_v_ characteristics at different voltages that may not be observed in the electrophysiological experiments. In addition to K_v_ characteristics at 50 mV, simulation models show that changes of amplitudes, time constants, and SSI with different voltages are consistent with previous studies (Ednie et al., 2019b).

Experimental results show that the proposed optimization method effectively calibrate simulation models of K_v_'s with *in-vitro* experimental data. These simulations models allow for the prediction of component K^+^ currents at different experimental conditions. In the Mgat1KO group, both I_Kto_ and I_Kslow_ densities are significantly reduced and the rate of I_Kslow_ inactivation is much slower, which are consistent with experimental observations.

This study couples simulation models with *in-vitro* experiments to investigate the effects of reduced N-glycosylation on K_v_ activity. A new method is used to delineate and examine multiple potassium currents. The computational simulation helps demonstrate the voltage-gating mechanism and conductance kinetics, thereby gaining a better understanding of glycosylation effects on K_v_ kinetics. This article does not include the 25s depolarization, which often helps distinguish I_Kslow1_ (K_v_1.5 activity) and I_Kslow2_ (K_v_2.1 activity). Our ongoing experiments focus on voltage-clamp experiments on 25-s periods. In the future work, new sets of *in-vitro* data will be analyzed via the *in-silico* modeling approach for the Mgat1KO effects on a variety of ionic currents.

## Data Availability Statement

The datasets generated and analyzed for this study are included in the article material and can be found in the GitHub repository https://github.com/haedong31/20_Kv_simulation.

## Ethics Statement

Animals were used and cared for as outlined by the NIH's Guide for the Care and Use of Laboratory Animals. All animal protocols were reviewed and approved by the Wright State University Institutional Animal Use and Care Committee.

## Author Contributions

HK: methodology, experimental analysis, and manuscript preparation. HY: methodology, experimental analysis, conceptualization, supervision, project administration, and manuscript preparation. AE and EB: data curation, clinical interpretation, and manuscript preparation. All authors contributed to the article and approved the submitted version.

## Funding

This work was supported in part by the National Science Foundation (MCB-1856132 to HY) and (MCB-1856199, IOS-1146882, and IOS-1660926 to EB), an American Heart Association, Greater Southeast Affiliate Grant-In-Aid (14GRNT20450148 to EB), and Postdoctoral Fellowship (15POST25710010 to AE). HK and HY would also like to thank the NSF I/UCRC Center for Health Organization Transformation (CHOT) award NSF IIP-1624727 for the support of their research work.

## Author Disclaimer

Any opinions, findings, or conclusions expressed in this article are those of the authors and do not necessarily reflect the views of the sponsor.

## Conflict of Interest

The authors declare that the research was conducted in the absence of any commercial or financial relationships that could be construed as a potential conflict of interest.

## Publisher's Note

All claims expressed in this article are solely those of the authors and do not necessarily represent those of their affiliated organizations, or those of the publisher, the editors and the reviewers. Any product that may be evaluated in this article, or claim that may be made by its manufacturer, is not guaranteed or endorsed by the publisher.

## Footnotes

^1^Mannosyl (α-1,3-)-Glycoprotein β-1,2-N-acetylglucosaminyltransferase.

^2^UDP-GlcNAc:α-3-D-mannoside-β1,2-N-acetylglucosaminyltransferase.
